# KRAS-mutation incidence and prognostic value are metastatic site-specific in lung adenocarcinoma: poor prognosis in patients with KRAS mutation and bone metastasis

**DOI:** 10.1038/srep39721

**Published:** 2017-01-04

**Authors:** Zoltan Lohinai, Thomas Klikovits, Judit Moldvay, Gyula Ostoros, Erzsebet Raso, Jozsef Timar, Katalin Fabian, Ilona Kovalszky, István Kenessey, Clemens Aigner, Ferenc Renyi-Vamos, Walter Klepetko, Balazs Dome, Balazs Hegedus

**Affiliations:** 1 National Koranyi Institute of Pulmonology, Budapest, Hungary; 2 Division of Thoracic Surgery, Department of Surgery, Comprehensive Cancer Center Vienna, Medical University of Vienna, Austria; 3 2nd Department of Pathology, Semmelweis University, Budapest, Hungary; 4 Molecular Oncology Research Group, Hungarian Academy of Sciences-Semmelweis University, Budapest, Hungary; 5 Department of Pulmonology, Semmelweis University, Budapest, Hungary; 6 1st Department of Pathology and Experimental Cancer Research, Semmelweis University, Budapest, Hungary; 7 Department of Thoracic Surgery, Ruhrlandklinik Essen, University Hospital of University Duisburg-Essen, Essen, Germany; 8 Department of Thoracic Surgery, National Institute of Oncology-Semmelweis University, Budapest, Hungary; 9 Department of Biomedical Imaging and Image-guided Therapy, Medical University of Vienna, Vienna, Austria

## Abstract

Current guidelines lack comprehensive information on the metastatic site-specific role of KRAS mutation in lung adenocarcinoma (LADC). We investigated the effect of KRAS mutation on overall survival (OS) in this setting. In our retrospective study, 500 consecutive Caucasian metastatic LADC patients with known KRAS mutational status were analyzed after excluding 32 patients with EGFR mutations. KRAS mutation incidence was 28.6%. The most frequent metastatic sites were lung (45.6%), bone (26.2%), adrenal gland (17.4%), brain (16.8%), pleura (15.6%) and liver (11%). Patients with intrapulmonary metastasis had significantly increased KRAS mutation frequency compared to those with extrapulmonary metastases (35% vs 26.5%, p = 0.0125). In contrast, pleural dissemination and liver involvement were associated with significantly decreased KRAS mutation incidence (vs all other metastatic sites; 17% (p < 0.001) and 16% (p = 0.02) vs 33%, respectively). Strikingly, we found a significant prognostic effect of KRAS status only in the bone metastatic subcohort (KRAS-wild-type vs KRAS-mutant; median OS 9.7 v 3.7 months; HR, 0.49; 95% CI, 0.31 to 0.79; p  = 0.003). Our study suggests that KRAS mutation frequency in LADC patients shows a metastatic site dependent variation and, moreover, that the presence of KRAS mutation is associated with significantly worse outcome in bone metastatic cases.

Oncogenic mutations of the Kirsten rat sarcoma viral oncogene homolog gene (KRAS) are frequently identified in lung, colorectal and pancreatic cancers^1^. In lung adenocarcinoma (LADC), the mutation rate was found to be up to 30%^2^,^3^,^4^,^5^. KRAS is a proto-oncogene that is a downstream member of the epidermal growth factor receptor (EGFR) signaling pathway. KRAS and EGFR activating mutations have been described to be usually mutually exclusive^6^.

While extensive data is available on the predictive and prognostic significance of KRAS mutation in colorectal carcinoma (CRC), we have ambiguous information on its prognostic role in lung cancer. In CRC, the presence of KRAS mutation is associated with increased metastatic potential and lack of treatment benefit from anti-EGFR monoclonal antibody therapy^7^,^8^. CRC patients with KRAS-mutant tumors have worse overall survival (OS) and increased incidence of lung, bone and brain metastasis^9^,^10^,^11^. Accordingly, Zou *et al*. found that KRAS mutations might be used as independent predictors of distant metastases in CRC^12^. Moreover, KRAS mutations in CRC patients were significantly associated with the number of pulmonary metastasis and with the lung as first site of recurrence after pulmonary metastasectomy^13^. Besides, KRAS mutation has been reported to be a potential negative prognostic factor in patients with liver metastatic colorectal cancer in various treatment modalities^14^,^15^,^16^.

Although the role of KRAS mutations in non-small cell lung cancer (NSCLC) is intensely investigated, there is limited and partly contradictory information on its prognostic role in lung cancer^5^,^17^,^18^,^19^. A recent pooled analysis including 1362 patients from four EGFR-TKI randomized controlled trials failed to show a survival difference in the placebo arm between patients with KRAS-mutated and wild-type tumors^19^. Moreover, our group has recently demonstrated that KRAS mutation status per se is not prognostic in platinum based chemotherapy treated unresected stage III–IV LADC^5^. On the other hand, several other studies including large meta-analyses demonstrated that KRAS mutation has a negative prognostic impact especially in early stage LADC^20^,^21^,^22^.

Beside its general prognostic role, the predictive value of KRAS mutational status in chemotherapy remains controversial^17^. Recently, a meta-analysis including 1677 advanced NSCLC patients revealed that patients with KRAS mutations had significantly lower ORR and potentially lower PFS after first-line chemotherapy^23^. Furthermore, the presence of KRAS mutations had a mild negative impact on OS in advanced NSCLC patients treated with first-line chemotherapy in 52 Italian institutions^24^. A retrospective analysis of 484 Asian advanced NSCLC patients showed only limited predictive role of KRAS mutation^25^. Additionally, several other studies failed to show a predictive role of KRAS mutation for first-line chemotherapy efficacy^26^,^27^,^28^.

Importantly, however, there is limited data available regarding the influence of KRAS mutation on the organ specificity of LADC metastases^29^,^30^,^31^. Nevertheless, KRAS mutation might have different prognostic or predictive role depending on the site of dissemination. For this very reason, the aim of our study was to investigate the metastatic site-specific prognostic value of KRAS mutation in LADC patients.

## Patients and Methods

### Study Population

In our retrospective analysis, 532 consecutive Caucasian patients with cytologically or histologically verified stage IV LADC were included at the National Koranyi Institute of Pulmonology and at the Department of Pulmonology, Semmelweis University between January 2009 and March 2013. All patients were (re)staged using the 7th edition of the TNM classification^32^. Age, Eastern Cooperative Oncology Group Performance Status (ECOG PS), smoking status, TNM stage and metastatic pattern was evaluated at the time of diagnosis. We differentiated single-organ and multiple-organ metastatic cases. Clinical follow-up was closed on the 1st of May, 2015. The study and all treatments were conducted in accordance with the current National Comprehensive Cancer Network guidelines, based on the ethical standards prescribed by the Helsinki Declaration of the World Medical Association and with the approval of the Scientific and Research Committee of the Hungarian Medical Research Council (52614–4/2013/EKU), which waived the need for individual informed consent for this retrospective study. Thirty-two patients with known EGFR mutations were excluded and the remaining 500 were analyzed within this study.

### Molecular testing

Based on the knowledge that KRAS, EGFR and ALK (anaplastic lymphoma kinase) mutations are mutually exclusive (with rare reported exceptions)^6^, in Hungary KRAS testing is performed at first in all adenocarcinoma or adenosquamous cases to exclude KRAS-mutant cases from EGFR analysis as part of a diagnostic algorithm. This screening strategy results in a large number of cases with KRAS mutation data.

All mutational analyses were performed at the 2^nd^ Department of Pathology and at the 1^st^ Department of Pathology and Experimental Cancer Research, Semmelweis University as previously described^33^. According to the contemporary National Comprehensive Cancer Network (NCCN) guideline^34^, DNA isolation was performed from formalin fixed paraffin-embedded (FFPE) tissue blocks or cytological specimens of primary tumors or lymphatic or organ metastases (including pleural effusion).

KRAS mutations were identified by microcapillary-based restriction fragment length analysis as described^5^. Briefly, tumor-rich microscopic area on H&E staining had been determined by pathologists prior to macrodissection from FFPE tissue or cytological smears. DNA was extracted using the MasterPure™ DNA Purification Kit (Epicentre Biotechnologies, WI) according to the instructions of the manufacturer. The microfluid-based restriction fragment detection system characterized by 5% mutant tumor cell content sensitivity. Density ratio of the mutated band to the WT one was calculated and samples containing >5% of the non-WT band were considered mutation positive due to the sensitivity threshold. The base-pair substitution in the mutant samples were verified and determined by sequencing on the ABI 3130 Genetic Analyzer System (Life Technologies, Carlsbad, CA) with the BigDye^®^ Terminator v1.1 Kit. EGFR mutation analysis was performed by Sanger Sequencing of the EGFR exon 18–21 as previously described^4^.

### Statistical Methods

Categorical parameters (gender (female vs male), smoking status (never- vs ever (former and current)-smoker, ECOG PS (0 vs 1), and KRAS mutation status (mutant vs wild-type)) of the patients cohorts with different organ specific metastatic pattern (lung, bone, adrenal, brain, pleura, and liver) were statistically analyzed by Chi-square test. Age as a continuous variable was analyzed in the different single-organ metastatic groups by Student’s t-test. Kaplan-Meier survival curves and two-sided log-rank tests were used for univariate survival analyses. The Cox proportional hazards model was used for univariate survival analyses to calculate the hazard ratios (HR) and corresponding 95% confidence intervals (CI). P values are always given as two-sided and were considered statistically significant below 0.05. Metric data is always shown as median or mean and corresponding range or, in case of OS, as median and corresponding 95% CI. All statistical analyses were performed using the PASW Statistics 18.0 package (Predictive Analytics Software, SPSS Inc., Chicago, IL, USA).

## Results

### Patient characteristics and metastatic pattern

Clinicopathological characteristics of patients with different metastatic pattern are shown in Table 1. We found 362 (72%) patients with single-organ metastatic disease and 138 (28%) cases with metastases affecting multiple organs (Table 1). The most frequent metastatic sites included lung (45.6%), bone (26.2%), adrenal gland (17.4%), brain (16.8%), pleura (15.6%), and liver (11%).

We did not find significant differences in age between the single-organ (62.3 ± 9.3) vs multiple-organ (60.8 ± 9.7) metastatic cohorts. Patients presented with only pleural spread (66.8 ± 10.4) were significantly older than those with only lung (62 ± 8.9), bone (60 ± 10.7), adrenal (63.1 ± 6.8) or brain (59.7 ± 9.2) metastases (p = 0.0024, p = 0.0008, p = 0.0132, p = 0.002, respectively). Patients with brain involvement were significantly younger than those with lung metastasis (p = 0.0094).

The proportion of patients with ECOG PS 0–1 was similar in the different organ-specific metastatic subgroups. Of note, we found significantly higher numbers of never-smokers in the subgroup of patients with pleural metastases (27%) when compared to all other sites (12.2%, p = 0.0018).

### Metastatic site-specific variation of KRAS status

**Figure 1:**
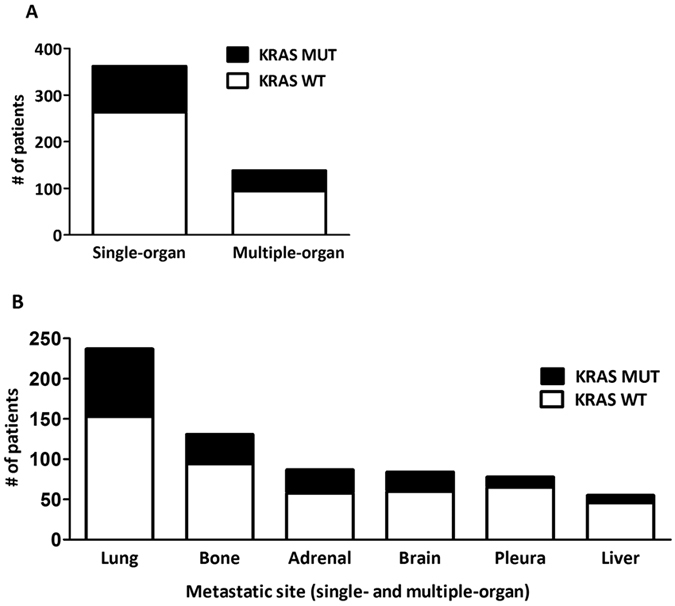
Metastatic site-specific variation of KRAS status. **(A)** Patients with multiple-organ metastases showed a non-significant increase in the percentage of KRAS mutant cases (vs single-organ spread, 32% v 27%). **(B)** In the organ-specific analysis, patients with brain, bone or adrenal gland metastases demonstrated similar KRAS mutation frequencies (29%, 28% and 33%, respectively). However, pulmonary metastatic cases demonstrated increased KRAS mutation frequency when compared to those with extrapulmonary metastases (35% and 26.5%; p = 0.0125). In contrast, pleural dissemination and liver metastasis associated with decreased KRAS mutation incidence (17% (p < 0.001) and 16% (p = 0.0023), respectively).

KRAS mutation incidence was 28.6% in the full cohort. Patients with multiple-organ metastases showed a non-significant increase in the percentage of KRAS mutation (vs single-organ spread, 32% vs 27%, Table 1, Fig. 1A). Metastatic site-specific variation of KRAS status is shown in Fig. 1. Importantly, patients with brain (29%), bone (28%) or adrenal gland (33%) metastases demonstrated similar KRAS mutation frequencies (Table 1, Fig. 1B). However, pulmonary metastatic cases demonstrated increased KRAS mutation frequency when compared to those with all extrapulmonary metastases (35% and 26.5%, p = 0.0125, Fig. 1B). In contrast, pleural dissemination and liver metastasis were associated with decreased KRAS mutation incidence (vs all other metastatic sites; 17% (p < 0.001) and 16% (p = 0.0023), respectively).

### Clinical Outcome

**Figure 2:**
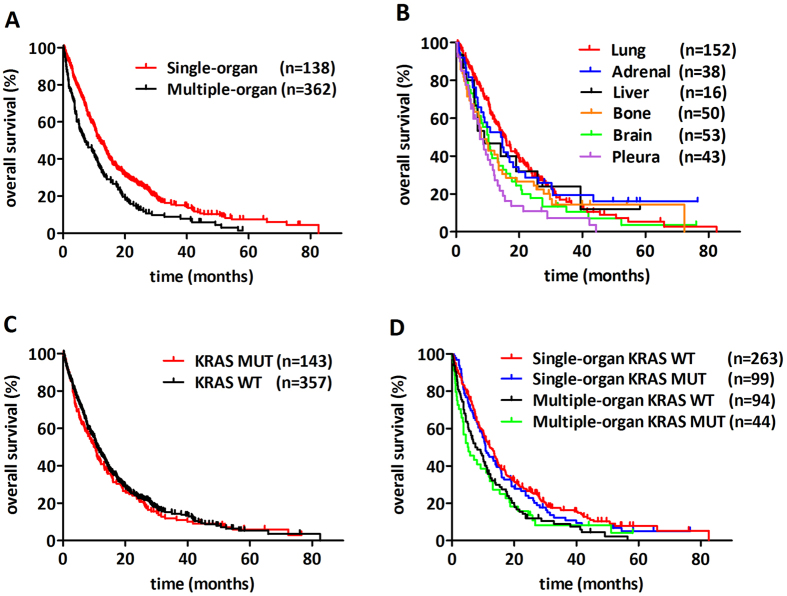
Kaplan-Meier curves for the OS of lung adenocarcinoma patients according to metastatic sites and KRAS mutation status. **(A)** Patients with multiple-organ metastases had significantly decreased median overall survival (OS) compared to those with single-organ metastasis (6.8 vs 11.6 months; HR, 0.62; 95% CI, 0.498 to 0.788; p < 0.001). **(B)** In the comparison of single-organ sites, patients presented with single-organ metastasis to the pleura demonstrated significantly decreased median OS when compared to those with lung (7.5 vs 15.6 months; HR, 0.460; 95% CI, 0.255 to 0.646; p < 0.001) or adrenal spread (7.5 vs 14.4 months; HR, 1.896; 95% CI, 1.154 to 3.114; p = 0.011). Furthermore, patients with brain metastasis showed significantly decreased OS when compared to patients presented with lung metastasis (median OS, 10.3 vs 15.6 months, respectively; HR, 1.5; 95% CI, 1.004 to 2.117; p = 0.04). We found no statistically significant information in any other organ specific comparison. KRAS mutation had no significant prognostic effect **(C)** in the full cohort of metastatic stage patients at diagnosis or according to **(D)** patients with single or multiple-organ spreads.

Patients with multiple-organ metastases had significantly decreased median overall survival (OS) compared to those with single-organ metastasis (6.8 vs 11.6 months, respectively; HR, 95% CI, 0.6262, 0.498 to 0.788, p < 0.001, Fig. 2A). Next, we compared the prognostic impact of single-organ metastatic sites (Fig. 2B). Patients with single-organ metastasis limited to the pleura demonstrated significantly decreased OS when compared to those with lung-only (median OS, 7.5 vs 15.6 months, HR, 0.460, 95% CI, 0.255 to 0.646; p < 0.001) or with adrenal-only spread (median OS, 7.5 vs 14.4 months, HR, 1.896, 95% CI, 1.154 to 3.114; p = 0.011). Furthermore, patients with brain metastasis showed significantly decreased OS when compared to patients presented with lung metastasis (median OS, 10.3 vs15.6 months, respectively; HR, 1.5; 95% CI, 1.004 to 2.117; p = 0.04). There was no statistically significant difference in other organ specific comparison.

Next, we investigated the impact of KRAS mutation status on OS in the metastatic cohort (Fig. 2C) including the comparison of multiple- and single-organ metastatic subgroups (Fig. 2D). Importantly, we found no statistically significant information in these comparisons.

**Figure 3:**
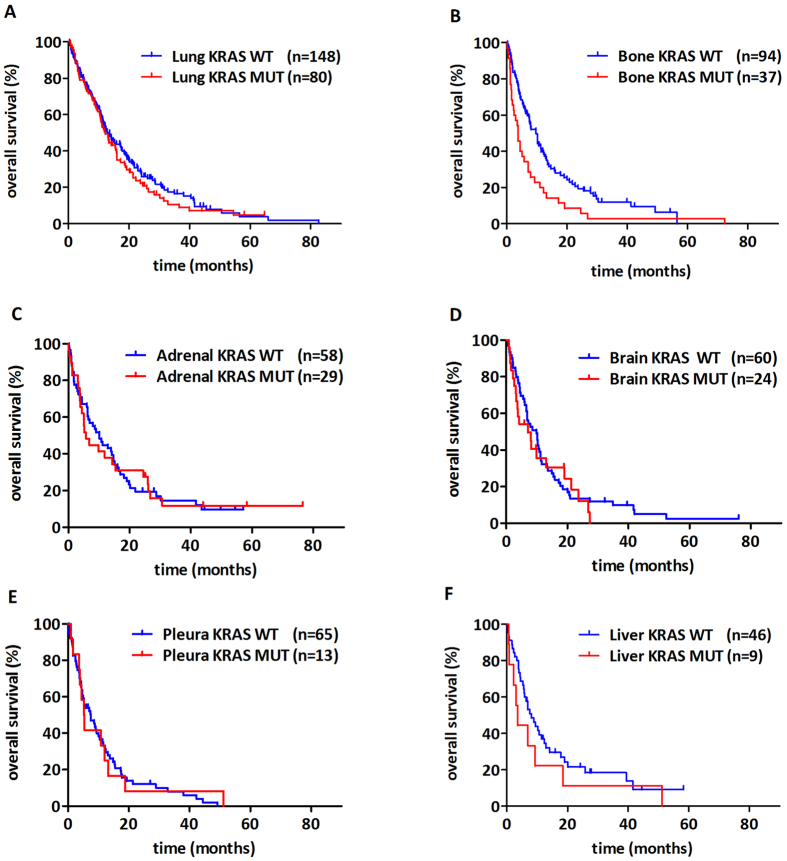
Kaplan-Meier curves for the OS of metastatic lung adenocarcinoma patients according to KRAS mutation status in patients with **(A)** lung, **(B)** bone, **(C)** adrenal, **(D)** brain, **(E)** pleura, and **(F)** liver spread. Both single- and multiple-organ metastatic cases were included in these analyses. We found a clinically relevant and also significant decrease in OS in patients presented with KRAS mutant bone metastasis (vs KRAS wild-type, median OS 3.7 v 9.7 months; HR, 0.49; 95% CI, 0.31 to 0.79; p = 0.003). Importantly, we found no statistically significant information in any other organ-specific comparison.

The impact of KRAS mutation status on OS of patients with different organ-specific metastases (including both multiple- and single-organ metastatic patients) is shown in Fig. 3. We observed a significant and clinically relevant decrease in OS in patients with KRAS-mutant tumors and with bone involvement (notably, this subcohort included bone metastastatic cases with or without non-skeletal metastasis) (vs those with KRAS-WT tumors and bone involvement; median OS 3.7 v 9.7 months, respectively; HR, 0.49; 95% CI, 0.31 to 0.79; p = 0.003; Fig. 3B). Of note, no further statistically significant differences were observed in any other organ-specific comparison. Moreover, KRAS-mutant lung adenocarcinoma patients with dissemination limited to the skeletal system (n = 13) tended to have a shorter OS then those with KRAS-WT tumors (7.0 vs 10.2 months; p = 0.21, Supplemental Fig. 1).

## Discussion

Despite of the extensive research, the prognostic and predictive power and thus the clinical utility of KRAS oncogenic mutations in lung adenocarcinoma has not yet been defined for over a decade^5^,^17^,^35^,^36^. Surprisingly, there is very limited comprehensive data available regarding the influence of KRAS mutation on the organ specificity of lung adenocarcinoma metastases^37^.

In line with other studies, we detected a significant decrease of median OS in patients with multiple-organ metastases^38^. This observation further supports the proposal that NSCLC staging should take into account the number and site of metastases since tumors with a single metastasis in a single organ had significantly better prognosis than those with multiple metastases in one or several organs^39^.

Comparing single-organ metastatic cases, we found that patients presented with metastasis to the pleura and brain showed significantly decreased OS when compared to patients exhibited lung metastasis. Earlier studies also showed that brain metastasis have an increased negative impact on survival^40^,^41^.

The KRAS mutation rate in the presented cohort (28.6%) is in line with other studies^17^,^42^. Patients with multiple-organ metastases showed a modest increase in the incidence of KRAS mutation. While there is no published data for lung adenocarcinoma, significantly increased frequency of KRAS mutation in patients with multiple organ metastases was found in a colorectal cancer study^43^. With regards to metastatic sites, in line with previous findings of Doebele *et al*.^37^, in our study patients with brain, bone or adrenal gland metastases demonstrated similar KRAS mutation frequencies. We detected 28% KRAS mutation frequency in the bone metastatic cohort which is similar to previous findings of Confavreux *et al*. and Bittner *et al*.^44^,^45^. However, we found pleural dissemination and liver metastasis associated with decreased and intrapulmonary with increased KRAS mutation incidence of the primary. Importantly, in accordance with our study, in colorectal cancer, RAS mutation was associated with increased lung^9^,^13^ and decreased liver metastatic capacity^43^,^46^. Nevertheless, further prospective studies are required to conclude if KRAS status can be used to risk stratify patients for the onset of pulmonary metastasis.

With regards to KRAS mutation status and the role of smoking in pulmonary metastasis, we found no association between metastatic pulmonary nodules and smoking. In previous studies, smoking was found not to be a significant risk factor in developing lung metastases in colorectal cancer either^47^,^48^,^49^. In contrast, in esophageal and breast cancer, smoking was reported to be associated with pulmonary spread^50^,^51^.

In line with other studies, we found a significant decrease of median OS in patients with multiple-organ metastases^38^. Our finding further supports the proposal that the M stage should take into account the number of metastases and the number of metastatic sites as tumors with a single metastasis in a single organ had significantly better prognosis than those with multiple metastases in one or several organs^39^.

To the best of our knowledge, this is the first study to directly compare the prognostic role of KRAS mutations in the distinct metastatic sites in lung adenocarcinoma. Importantly, we demonstrated a significant and also clinically relevant decrease in the OS of patients with KRAS-mutant lung adenocarcinoma and bone metastasis. The differences between the clinicopathological characteristics of KRAS-WT and KRAS-mutant bone-metastatic patients cannot explain the observed decrease in OS. Of note, we found higher frequency of multiple-organ metastases in KRAS-WT patients presenting with bone metastases when compared to KRAS-mutant cases (84% v46%, p < 0.0001).

Our study has several limitations, in part due to its retrospective nature. Although we excluded EGFR mutants from our study, the KRAS-WT cohort was not analyzed for additional oncogenic driver mutations. In addition, reflecting the routine clinical practice the majority of patients are evaluated by clinical and not pathological TNM staging. Accordingly, we are not able to exclude the presence of asymptomatic disease or micro metastases. It is important to mention that in the case of the relatively less frequent metastatic site with the lowest KRAS mutation incidence, namely in the liver metastasis subgroup, we do not have sufficient statistical power to determine the impact of KRAS mutation on overall survival.

Despite enormous attempts the prognostic value and the clinical utility of the most frequently occurring oncogene have not been established in advanced stage lung adenocarcinoma for over a decade. Consequently, guidelines lack information on the clinical benefit of KRAS mutation testing in NSCLC. Therefore, and more importantly, our study addresses an important issue and highlights the possible prognostic importance and potential clinical relevance of KRAS mutation. In addition, our study is the first that showed metastatic site-specific variation of the prognostic value of KRAS status in lung adenocarcinoma. We suggest the KRAS mutation status may have important implications for diagnostic strategies and treatment decisions. Our results indicate that KRAS mutation status has a strong prognostic value in bone metastatic lung adenocarcinoma patients. Nevertheless, additional studies are needed to evaluate the ability of KRAS mutation analysis to risk stratify patients with bone metastasis. Future studies are also required to investigate whether KRAS status might even predict response to various treatment options for bone metastatic patients.

## Additional Information

**How to cite this article**: Lohinai, Z. *et al*. KRAS-mutation incidence and prognostic value are metastatic site-specific in lung adenocarcinoma: poor prognosis in patients with KRAS-mutation and bone metastasis. *Sci. Rep.* **7**, 39721; doi: 10.1038/srep39721 (2017).

**Publisher's note:** Springer Nature remains neutral with regard to jurisdictional claims in published maps and institutional affiliations.

## Supplementary Material

Supplementary Information

## Figures and Tables

**Table 1:** Clinicopathological characteristics, overall survival (OS), KRAS mutational status and metastatic pattern in lung adenocarcinoma patients.

Metastatic site		Total	Single-organ	Multiple-organ	Lung*	Bone*	Adrenal*	Brain*	Pleura*	Liver*
Number of patients		500	362**	138	228	131	87	84	78	55
Single-organ only					152	50	38	53	43	16
Age (mean ± SD)		61.9 ± 9.4	62.3 ± 9.3	60.8 ± 9.7	62 ± 8.9	60 ± 10.7	63.1 ± 6.8	59.7 ± 9.2	66.8 ± 10.4	64.4 ± 9.1
Gender	Male	245 (49%)	181 (50%)	64 (46%)	102 (45%)	74 (56%)	34 (39%)	36 (43%)	38 (49%)	26 (47%)
	Female	255 (51%)	181 (50%)	74 (54%)	126 (55%)	57 (44%)	53 (61%)	48 (57%)	40 (51%)	29 (53%)
ECOG PS	0–1	459 (94%)	335 (94%)	124 (92%)	218 (97%)	115 (91%)	75 (87%)	77 (93%)	71 (91%)	48 (91%)
	>1	32 (6%)	21 (6%)	11 (8%)	7 (3%)	11 (9%)	11 (13%)	6 (7%)	7 (9%)	5 (9%)
	Unknown	9	6	3	3	5	1	1	0	2
Smoking	Never	67 (15%)	52 (16%)	15 (12%)	29 (14%)	18 (16%)	7 (9%)	7 (9%)	20 (27%)	4 (8%)
	Former	141 (31%)	104 (31%)	37 (30%)	61 (29%)	32 (28%)	20 (25%)	25 (32%)	23 (32%)	20 (41%)
	Current	250 (54%)	179 (53%)	71 (58%)	117 (57%)	65 (58%)	52 (66%)	45 (58%)	30 (41%)	25 (51%)
	Unknown	42	27	15	21	16	8	7	5	6
KRAS	Wild-type	357 (71%)	263 (73%)	94 (68%)	148 (65%)	94 (72%)	58 (67%)	60 (71%)	65 (83%)	46 (84%)
	Mutant	143 (29%)	99 (27%)	44 (32%)	80 (35%)	37 (28%)	29 (33%)	24 (29%)	13 (17%)	9 (16%)
***Median OS (months)		10.8	11.67	6.87	15.6***	7.9***	14.4***	10.3***	8.8***	8.9***

*Values include both single- and multiple-organ cases except for OS. **In 10 cases, other than the listed types of metastasis was present (renal and subcutaneous). ***Only single-organ metastatic patients were included. Data shown in parentheses are column percentages; ECOG PS, Eastern Cooperative Oncology Group performance status; OS, overall survival.
