# Supplementary material for: KRAS-mutation incidence and prognostic value are metastatic site-specific in lung adenocarcinoma: poor prognosis in patients with KRAS mutation and bone metastasis
Source: Sci Rep. 2017 Jan 4;7:39721. doi: 10.1038/srep39721 (PMC5209707; doi:10.1038/srep39721)

Supplemental figure 1 (online only)

KRAS-mutation incidence and prognostic value are metastatic site-specific in lung adenocarcinoma: poor prognosis in patients with KRAS mutation and bone metastasis

Zoltan Lohinai, MD, Thomas Klikovits, MD, Judit Moldvay, MD, PhD, Gyula Ostoros MD, PhD, Erzsebet Raso, PhD, Jozsef Timar, MD, PhD, Dsc, Katalin Fabian, MD, Ilona Kovalszky, MD, PhD, István Kenessey, MD, PhD, Clemens Aigner, MD[2], Ferenc Renyi-Vamos MD, PhD, Walter Klepetko, MD, Balazs Dome, MD, PhD, Balazs Hegedus, PhD

e-Figure 1. Kaplan-Meier curves for the OS in metastatic lung adenocarcinoma patients according to KRAS mutation status in patients with single-organ bone spread. We found a non-significant decrease in median OS in patients presented with KRAS mutant bone metastasis (vs KRAS wild-type; 7.0 vs 10.2 months; log-rank test).

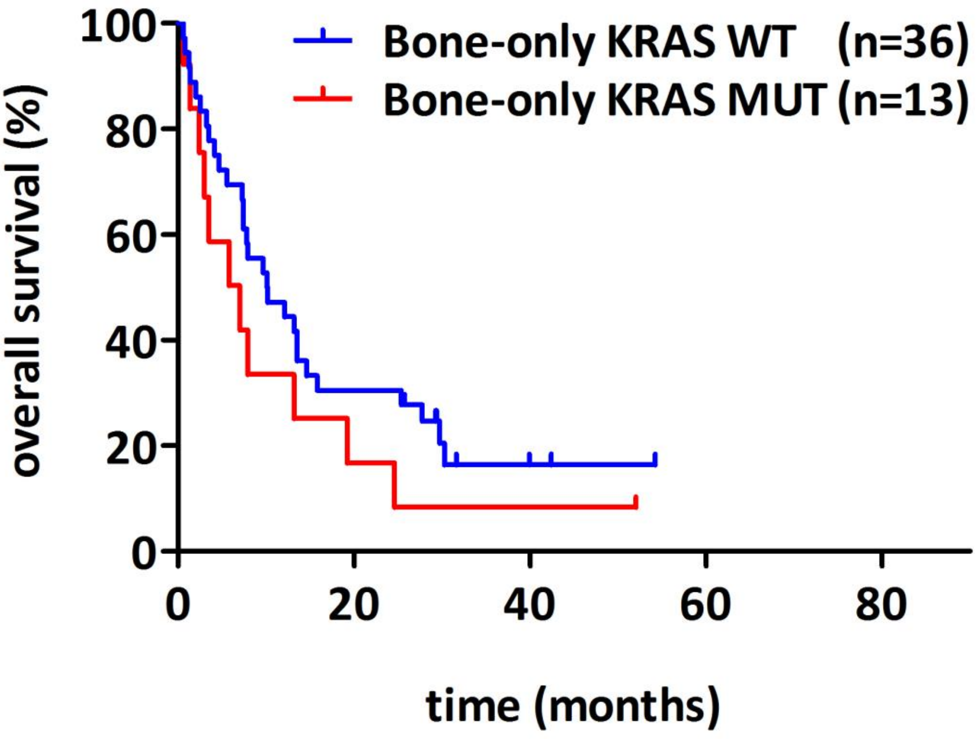

Supplement: Supplementary Information [file srep39721-s1.pdf]
